# Effects of Rumen-Protected Choline on Growth Performance, Carcass Characteristics and Blood Lipid Metabolites of Feedlot Lambs

**DOI:** 10.3390/ani10091580

**Published:** 2020-09-04

**Authors:** Jorge R. Kawas, Jose F. Garcia-Mazcorro, Hector Fimbres-Durazo, Maria E. Ortega-Cerrilla

**Affiliations:** 1 Facultad de Agronomia, Universidad Autonoma de Nuevo Leon, General Escobedo 66050, Mexico; 2 Research and Development, MNA de Mexico, San Nicolas de los Garza 66477, Mexico; josegarcia_mex@hotmail.com; 3 Facultad de Medicina Veterinaria y Zootecnia, Universidad Autonoma de Nuevo Leon, General Escobedo 66050, Mexico; hfimbres@hotmail.com; 4 Programa de Ganadería, Colegio de Postgraduados, Montecillo 56230, Mexico; faikma@yahoo.com.mx

**Keywords:** choline, rumen-protected choline, feedlot lambs, lipid metabolism

## Abstract

**Simple Summary:**

Choline is important for animal health, due to its involvement in the synthesis of vital molecules in the body. Several feed materials used in animal nutrition contain choline, but this naturally occurring choline is rapidly degraded in the rumen, therefore, it should be offered as rumen-protected choline (RPC) in ruminant animal species. Here we describe the results of a study that we performed with the aim of evaluating the effect of RPC on growth, carcass, and some blood metabolites in feedlot lambs. RPC supplementation did not significantly affect dry-matter intake, weight gain, gain:feed ratio, or carcass weights. Interestingly, RPC supplementation was associated with lower blood triglycerides and increased backfat thickness and yield grade, thus suggesting an effect of RPC on lipid metabolism. RPC supplementation was also associated with a reduced height to the shoulder and longissimus muscle area, suggesting an inhibitory effect of RPC on growth. The results of this study do not support the use of RPC supplementation to improve animal performance or carcass characteristics in feedlot lambs.

**Abstract:**

Choline is an essential nutrient for animals, but dietary choline is degraded in the rumen, and thus, should be offered as rumen-protected choline (RPC) in ruminants. In this article, we investigate the effect of RPC supplementation in feedlot lambs. Forty intact male Saint Croix lambs (average: 20.3 kg, 3–4 months of age) on a high grain-low roughage base feed were randomly assigned to four treatments (0, 0.1, 0.2, and 0.3% RPC on dry-matter basis; *n* = 10 per group). RPC was offered for 90 days after 15 days of adaptation. RPC supplementation was not associated with significant differences in dry matter intake, weight gain, gain:feed ratio, carcass weights, and the dressing percentages. There was a linear decrease in height to the shoulder (*p* = 0.013) and longissimus muscle area (*p* = 0.051) with higher RPC levels, and a higher backfat thickness and yield grade with 0.3% RPC compared to 0.1% RPC (*p* < 0.05). Blood triglycerides concentrations were higher in control (0% RPC) compared to 0.3% RPC (*p* = 0.008). The lack of significant effects on growth performance and the results on backfat thickness and yield grade, may indicate undesirable effects associated with RPC supplementation. More research is needed to establish the needs and specific quantities of RPC supplementation in feedlot lambs.

## 1. Introduction

Diet is one of the most important factors affecting animal performance in production systems. Choline is a water-soluble essential nutrient for animals that is often not classified as a vitamin because it is required in gram rather than milligram amounts [1,2,3]. Choline is a key compound for the synthesis of vital molecules in the body, such as phosphatidylcholine and acetylcholine. Phosphatidylcholines are the most abundant phospholipids in animal tissues, and they are involved in lipid absorption and transport, as well as the synthesis of lipoproteins [4], while acetylcholine is a neurotransmitter that plays a critical role in muscle contractions and brain activity [5]. 

The requirements of choline in farm animals have not been well established because of several reasons. First, feed materials (e.g., maize) contain different concentrations of naturally occurring choline that display various levels of bioavailability, and this choline in feed interacts with other substances (e.g., methionine, cobalamin, and folic acid) involved in transmethylation reactions [6]. Also, the combination of different levels and composition of fat, protein, and carbohydrates in the diet as well as the age, caloric intake, and growth rate of animals have an influence on the lipotropic action of choline, and thus, the requirement of this nutrient [7]. Furthermore, naturally occurring choline in feed is rapidly degraded by the rumen microbiota, where its N-methyl groups are converted into trimethylamine, and ultimately, methane [8,9,10,11,12]. Thus, even choline-rich feedstuffs can only marginally contribute to the post-ruminal supply of choline, and choline supplementation should, therefore, be provided as rumen-protected choline (RPC) in ruminant animal species.

Despite the difficulties in establishing the requirements of choline in farm animals, growing evidence suggests that ruminant species may experience a choline deficiency and that production may be increased with supplementation [3]. Also, RPC supplementation has been shown to reduce the accumulation of liver lipids and improve milk production in lactating cows [13,14,15], increase weight gain and carcass characteristics in beef cattle [16,17,18], as well as promote growth performance, lower fat content in tissues and improve meat quality in lambs [19,20]. The exact mechanism explaining the relationship between RPC supplementation, growth, and carcass characteristics is unclear at the moment, but likely relates to methionine sparing [21] and/or modulation of lipid metabolism [17]. However, this phenomenon may differ widely between breeds and animal species [22,23,24]. In the lamb, the synthesis of extra-hepatic choline and the reutilization of biliary choline between the intestine and the liver contribute to the maintenance of a greater body reserve of endogenous choline in this species [25,26,27]. Also, feedlot diets with high proportions of rapidly fermentable carbohydrates lead to low rumen pH, which impacts the equilibrium of microorganisms within the rumen [28], and therefore, the degradation of choline and other nutrients. The objective of this study was to determine the effect of RPC supplementation on growth performance, carcass characteristics, and blood lipid metabolites of feedlot lambs. 

## 2. Materials and Methods 

This study was conducted in compliance with the current Mexican legislation (NOM-062-ZOO-1999) and revised by the Committee of Animal Research and Experimentation (CARE) at MNA de Mexico (Protocol # 15012018). 

### 2.1. Animals and Experimental Design

Forty intact male Saint Croix lambs (3 to 4 months of age, 20.3 kg average body weight) were purchased from a local sheep producer, transported to our facilities in the municipality of Higueras, Nuevo León, México, and randomly assigned to four treatments (0, 250, 500, and 750 mg RPC/day, *n* = 10 per group) that were equivalent to 0, 0.1, 0.2, and 0.3% RPC on dry-matter (DM) basis. These doses were chosen based on previous literature [3,17,20] and considering cost-efficiency in lamb feedlots. A high grain-low roughage base feed with 17.6% crude protein (Table 1) was used to prepare the experimental diets [3,29]. Diets were offered two times daily at 8:00 h and 17:00 h. RPC supplementation was offered for 90 days following a 15-day adaptation period. The RPC source had a minimum concentration of choline chloride of 25% (ReaShure^®^, Balchem Corp., Slate Hill, NY, USA). The lambs were individually confined to 1 × 2 m shaded pens equipped with water and feed troughs. The lambs were dewormed using ivermectin (Ivomec, Merial de Mexico, Queretaro, Mexico) and closantel (Closantil, Chinoin, Mexico City, Mexico), vaccinated against clostridial disease (Covexin 10, Schering Plough Animal Health, Kenilworth, NJ, USA), and supplemented with vitamins (Virbamec^®^ ADE, Virbac Mexico, Guadalajara, Mexico). Fresh clean water was offered ad libitum.

### 2.2. Feed Analyses

A 500 g composite sample of offered feed was ground through a 2 mm screen in a Wiley mill. Concentrations of DM, crude protein (CP), ether extract (EE), and ash were analyzed using official methods of analysis from the Association of Official Analytical Chemists (AOAC, 2019) [30]. Neutral detergent fiber (NDF) was determined using filtration bags and a fiber analyzer (Model A200, ANKOM Technology, Macedon, NY, USA). Non-fibrous carbohydrate (NFC) content was calculated using this formula:

NFC = DM − (CP + EE + Ash + NDF).(1)

### 2.3. Growth Performance and Body Measurements

We evaluated dry matter intake (g/d), dry matter intake adjusted for metabolic weight (g/kg^0.75^), daily weight gain, and gain:feed ratios. Feed offered was based on the intake of the previous day plus an additional 10% in order to reduce the selection of feed components, and feed offered, and orts collected before the morning meal were recorded daily to calculate the feed intake. Growth performance and blood lipid metabolites were evaluated by period (period 1: 0–45 days; period 2: 45–90 days). At the end of the experiment (day 90 of RPC supplementation), height to the shoulder, body length, and chest circumference were measured for all lambs before they were transported to the slaughterhouse. The carcasses were weighed and stored at −5 °C for 24 h. The hot and chilled carcass weights were recorded, and the dressing percent (warm carcass weight divided by the shrunk live weight) was calculated. Blood, visceral organ and kidney, pelvic, and heart fat weights were recorded. The backfat thickness and the longissimus muscle area between the 12th and 13th ribs were measured. The yield grade was calculated using this formula [31]:

yield grade = 0.4 + (10 × backfat thickness, in cm)(2)

### 2.4. Serum Triglycerides and Cholesterol Concentrations 

On days 45 and 90 of RPC supplementation, blood samples (5 mL) were collected before the morning meal (between 7:00 and 8:00 h) via jugular venipuncture (silicone-coated tubes without anticoagulant; BD Vacutainer^®^, Becton, Dickinson and Company, Franklin Lakes, NY, USA). The samples were left to clot for 30 min at ambient temperature and then centrifuged at 1000× *g* for 15 min. The blood serum was separated and stored at −80 °C until analysis for triglyceride and cholesterol concentrations (VetTest^®^ Analyzer, Idexx Laboratories, Westbrook, ME, USA).

### 2.5. Statistical Analysis

Dry matter intake, average daily gain, gain:feed, and blood lipid metabolites, were analyzed with the MIXED procedure using SAS v.9.04 in SAS University Edition. The statistical model included period, RPC level and their interaction as fixed effects. Body measurements, organ weights, and carcass characteristics were analyzed with the GLM procedure for linear and quadratic responses to the RPC level using orthogonal contrasts. The Tukey’s test was used for multiple comparisons. For variables measured at 45 and 90 days (intake daily gains, gain:feed ratio, serum triglycerides, and cholesterol), we used a repeated measures analysis. A *p* < 0.05 was considered to determine statistical significance in the comparisons. 

## 3. Results

### 3.1. Feeding and Growth Parameters

Initial body weights did not differ among treatments (*p* = 0.969). There was no significant difference in dry matter intake (DMI) and gain:feed among treatments, but there was a trend for statistically significant difference in average daily gain (ADG) (*p* = 0.061, Table 2). DMI, ADG, and gain:feed were higher during the second period (45–90 days), but the adjusted DMI (g/kg^0.75^) was lower during this same period (Table 2). Significant interactions between period and treatment were found for ADG and gain:feed, meaning that the study period affected the response to different RPC levels (Table 2). 

### 3.2. Body Measurements

There was no significant effect of RPC supplementation on body measurements and visceral body weights, with the exception of height to the shoulder that showed a linear trend to decrease with increased RPC levels (Table 3). The hot and chilled carcass weights, and the dressing percentages, were also not affected by RPC supplementation (Table 4). The longissimus muscle area was reduced linearly (*p* = 0.051) from 13.3 to 11.8 cm^2^ as the RPC level increased in the diet (Table 4). Backfat thickness showed a linear increase (*p* = 0.014) and was significantly higher, with 0.3% compared to 0.1% RPC (*p* < 0.05) (Table 4). Yield grade was also significantly higher, with 0.3% compared to 0.1% RPC (Table 4).

### 3.3. Serum Triglycerides and Cholesterol Concentrations

Serum cholesterol concentrations were not different among treatments (*p* = 0.432), but were lower at day 45 (38.4 mg/dL) compared to day 90 (56.9 mg/dL) (Table 5); however, these values are still considered to be within normal ranges (44–90 mg/dL). In contrast, there was a significant difference in serum triglycerides among the treatments (*p* = 0.007) with a clear trend of lower triglycerides concentrations with higher RPC levels (Table 5). 

## 4. Discussion

Choline is an essential nutrient with relevant functions in animal metabolism, and several feed materials contain measurable amounts of naturally occurring choline. However, dietary choline in ruminant species is rapidly degraded in the rumen, and thus, should be offered as RPC to fulfill the requirements of this nutrient. The requirements of choline in farm animals are not well established, but growing evidence suggests that RPC supplementation can increase animal productivity [3], although this differs widely between production systems, animal species, and breeds. For instance, significant amounts of choline are synthesized in extra-hepatic tissues of lambs and are extensively re-circulated between the intestinal tract and the liver [25,26,27]. It is unknown if other small ruminants have the same ability to recycle choline, but responses to RPC or betaine, an oxidative product of choline from liver and kidney metabolism, are thought to be comparable between sheep and goats [3].

This study showed that RPC supplementation was not associated with differences in growth performance, and this is interesting in the context of small ruminant nutrition. In a similar study, Li et al. [20] investigated the effects of RPC supplementation (0, 0.25, 0.50, and 0.75% for 60 days) on growth performance, meat quality, and gene expression in the longissimus muscle of Dorper x Hu lambs consuming a diet with 12% CP. In their study, lambs with 0.25% RPC showed statistically higher ADG (211 g) compared to animals with 0% (186 g), 0.5% (178 g) and 0.75% (170 g) RPC. In contrast, our study only showed a trend for significance in ADG among the treatment groups (lowest: 182 g with 0.2% RPC; highest: 226 g with 0.3% RPC). Key differences between our study and the study published by Li et al. [20] include the different levels of RPC supplementation and a higher percentage of protein in our diets (17.6% vs. 12%), which may have supplied an excess of methyl donors as substrates for choline synthesis by rumen microorganisms. Also, we used diets with more grain and less fiber compared to the diets offered by Li et al. [20], which can inevitably alter the ruminal microbiota, and therefore, differently modulate the metabolism of choline and other nutrients. Another key difference between the two studies is related to the animals’ breed, an often-neglected factor that has been shown to be associated with a different rumen microbiome and feed efficiency in other ruminant species [32].

Another area of interest is the effect of RPC on body measurements, muscle, and fat metabolism. Choline is often referred to as a lipotropic factor in dairy cattle (the animal species where choline has been most widely studied) because it prevents the abnormal accumulation of fat in the liver by promoting its transport or by increasing the utilization of fatty acids. In lambs, Li et al. [20] showed that the expression of several genes related to lipogenesis varied between the RPC levels, but there were no significant changes in dressing percentage and intramuscular fat. In comparison, in this study, we showed little effects on dressing percentages, but statistically significant differences in backfat thickness, and consequently, in yield grade (calculated using backfat thickness—the lower the yield grade, the better, according to the USDA scale). Another more recent study in Hu sheep showed lower abdominal fat with 2.2 g/d rumen-protected betaine (RPB), increased fat contents in longissimus dorsi with increasing RPB levels, and upregulation of *PI3K*, *mTOR*, and *S6K1* [33], genes that have been shown to be affected by the level of nutrient intake in cattle [34]. On the other hand, Li et al. [20] showed that RPC supplementations had no significant effect on triglycerides and cholesterol, and another study with wether goats also showed that RPC supplementation had no effect on triglycerides [35]. In contrast, in this study, we showed a linear decrease in serum triglycerides with increased RPC levels, perhaps indicating biological differences in fatty acid mobilization between breeds and species of small ruminants. The higher blood concentration of cholesterol during the second period may be explained by a differential tissue requirement during growth [36,37], although this varies among the different breeds [38]. This is important when considering dietary supplementation with choline and other nutrients that affect lipid metabolism at different developmental stages [39].

The effect of RPC on growth and productive performance, as well as lipid metabolism and muscle development, depends on the rumen passing rate and bioavailability of RPC and not only on the dose. However, to our knowledge, this phenomenon has not been investigated in small ruminants. Interestingly, one study in lactating cows suggests that the net portal flux of free choline was low (13%) after RPC dietary supplementation compared to the abomasal delivery of choline (61%) [40]. This is an important topic that deserves more attention for a better assessment of the effect of RPC supplementation in diets for ruminants.

## 5. Conclusions

In conclusion, our results show that RPC supplementation did not affect productive performance and carcass characteristics of feedlot lambs. However, the results on height to the shoulder, longissimus muscle area, and backfat thickness, suggest a negative impact on growth. More studies are necessary to investigate the relationship between impaired growth and changes in blood lipid metabolites during RPC supplementation in feedlot lambs. 

## Figures and Tables

**Table 1:** Base feed composition used to prepare the experimental diets.^1^

Ingredient, as Fed Basis	Percent
Soybean hulls	5.0
Sorghum, grain ^2^	69.5
Soybean meal	16.5
Cane molasses	6.0
Calcium carbonate	0.9
Urea	0.5
Salt	1.0
Premix ^3^	0.6
**Chemical Composition, Dry Basis**	
Crude protein	17.6
Neutral detergent fiber	15.8
Ether extract	2.5
Ash	2.4
Metabolizable Energy (Mcal/kg) ^4^	2.5

^1^ Rumen protected choline at a concentration of 25% (Capshure; Balchem Corporation, Slate Hill, NY), substituted 0, 0.1, 0.2 and 0.3% of sorghum in the respective experimental diets. ^2^ Sorghum grain: 50% whole and 50% ground. ^3^ Premix: Ammonium chloride (500 g/kg), trace minerals (Fe, 4000 mg/kg; Mn, 4800 mg/kg; Zn, 5460 mg/kg; Cu, 1000 mg/kg; I, 140 mg/kg; Co, 16.6 mg/kg; Se, 16.4 mg/kg); vitamin A (12,000,000 I.U./ton) and E (1,200,000 I.U./ton), sodium lasalocid (25 mg/kg). ^4^ Metabolizable energy based on values for each ingredient.

**Table 2:** Intake, weight gain, and gain:feed of lambs fed finishing diets containing various rumen-protected choline (RPC) levels.

	Period (days)			RPC Level (%) ^1^					*p* Values		
	0–45	45–90	S.E.	0	0.1	0.2	0.3	S.E.	Period	RPC	Period*RPC
Dry matter intake											
g/d	929	1085	25.0	1027	1007	964	1030	35.4	0.001	0.530	0.405
g/kg^0.75^	65.7	59.8	1.2	63.2	63.3	62.2	62.4	1.7	0.001	0.955	0.372
Average daily gain (g/d)	171	249	8.9	209	222	182	226	12.5	0.001	0.061	0.007
Gain:feed (g/g)	0.182	0.234	0.009	0.205	0.217	0.191	0.220	0.013	0.001	0.371	0.004

^1^ Means with different superscript letters indicate statistical significance (*p* < 0.05). S.E., standard error.

**Table 3:** Body measurements and visceral organ weights of lambs fed finishing diets containing various RPC levels.

Variable	RPC Level (%) ^1^					*p* Values	
	0	0.1	0.2	0.3	S.E.	Linear	Quadratic
Height to the shoulder (cm)	49.0	49.9	47.2	46.4	0.807	0.013	0.664
Body length (cm)	61.1	60.7	58.5	60.0	1.017	0.182	0.307
Chest circumference (cm)	79.9	81.9	79.9	81.7	1.220	0.467	0.973
Skin (kg)	5.1	5.1	4.8	5.2	0.190	0.950	0.789
Weight of visceral organs (kg)							
Liver	0.703	0.785	0.682	0.758	0.039	0.668	0.925
Kidney	0.102	0.108	0.096	0.105	0.004	0.950	0.789
Heart	0.155	0.168	0.171	0.167	0.007	0.219	0.284
Lung	0.666	0.702	0.601	0.665	0.033	0.463	0.656
Blood	1.166	1.324	1.272	1.346	0.075	0.151	0.573

^1^ Means with different superscript letters indicate statistical significance (*p* < 0.05). S.E., standard error.

**Table 4:** Slaughter weight, and hot and chilled carcass dressing percentages, of lambs that were fed rations containing various RPC levels.

Carcass Characteristics	RPC Level (%) ^1^					*p* Values	
	0	0.1	0.2	0.3	S.E.	Linear	Quadratic
Slaughter weight (kg)	39.1	40.1	37.1	40.1	1.00	0.976	0.328
Hot carcass weight (kg)	19.5	19.9	18.4	19.8	0.55	0.853	0.382
Chilled carcass weight (kg)	18.8	19.2	17.5	19.3	0.50	0.881	0.182
Hot carcass dressing (%)	49.8	49.6	49.5	49.4	0.94	0.794	0.973
Chilled carcass dressing (%)	48.1	48.0	47.1	48.1	0.74	0.813	0.445
Backfat thickness (mm)	0.621 ^a,b^	0.356 ^b^	0.787 ^a,b^	1.880 ^a^	0.014	0.014	0.068
Longissimus muscle area (cm^2^)	13.3	13.0	12.5	11.8	0.55	0.051	0.732
KPH fat (kg) ^2^	1.229	1.397	1.223	1.358	0.147	0.745	0.912
KPH fat (% of carcass weight) ^2^	6.3	7.1	6.6	6.8	0.66	0.730	0.685
Yield grade	0.64 ^a,b^	0.54 ^b^	0.71 ^a,b^	1.14 ^a^	0.14	0.140	0.068

^1^ Means with different superscript letters indicate statistical significance (*p* < 0.05). ^2^ KPH, kidney, pelvic, and heart fat. S.E., standard error.

**Table 5:** Blood serum triglyceride and cholesterol concentrations of lambs fed diets containing various RPC levels.

Blood Metabolites	Period (days)			RPC Level (%) ^1^					*p* Values		
	45	90	S.E.	0	0.1	0.2	0.3	S.E.	Period	RPC	Period*RPC
Triglycerides (mg/dL)	14.2	12.9	1.40	19.3 ^a^	13.6 ^a,b^	12.3 ^a,b^	9.0 ^b^	1.97	0.515	0.007	0.844
Cholesterol (mg/dL)	38.4	56.9	2.58	49.7	47.5	42.8	42.6	3.65	0.002	0.432	0.911

^1^ Means with different superscript letters indicate statistical significance (*p* < 0.05). S.E., standard error.

## References

[1] Huber J.T., Church D.C. (1988). Vitamins in ruminant nutrition. The Ruminant Animal Digestive Physiology and Nutrition.

[2] Zeisel S.H., Holmes-McNary M., Rucker R.B., Suttie J.W., McCormick D.B., Machlin L.J. (2001). Choline. Handbook of Vitamins.

[3] National Research Council of the National Academies, Committee on the Nutrient Requirements of Small Ruminants, Board on Agriculture and Natural Resources, Division on Earth and Life Studies (2007). Vitamins. Nutrient Requirements of Small Ruminants: Sheep, Goats, Cervids, and New wWrld Camelids.

[4] Lagace T.A. (2015). Phosphatidylcholine: Greasing the cholesterol transport machinery. Lipid Insights.

[5] Kuo I.Y., Ehrlich B.E. (2015). Signaling in muscle contraction. Cold Spring Harb. Perspect. Biol..

[6] (2011). EFSA Panel on Additives and Products or Substances used in Animal Feed (FEEDAP) Scientific Opinion on safety and efficacy of choline chloride as a feed additive for all animal species. EFSA J..

[7] Mookerjea S. (1971). Action of choline in lipoprotein metabolism. Fed. Proc..

[8] Broad T.E., Dawson R.M.C. (1976). Role of choline in the nutrition of the rumen protozon *Entodinium caudatum*. J. Gen. Microbol..

[9] Neill A.R., Grime D.W., Dawson R.M.C. (1978). Conversion of choline methyl groups through trimethylamine into methane in the rumen. Biochem. J..

[10] Neill A.R., Grime D.W., Snoswell A.M., Northrop A.J., Lindsay D.B., Dawson R.M. (1979). The low availability of dietary choline for the nutrition of the sheep. Biochem. J..

[11] Sharma B.K., Erdman R.A. (1988). Effects of high amounts of dietary choline supplementation on duodenal choline flow and production responses of dairy cows. J. Dairy Sci..

[12] Sharma B.K., Erdman R.A. (1989). In vitro degradation of choline from selected feedstuffs and choline supplements. J. Dairy Sci..

[13] Erdman R.A., Sharma R.A. (1991). Effect of dietary rumen-protected choline in lactating dairy cows. J. Dairy Sci..

[14] Pinotti L., Baldi A., Politis I., Rebucci R., Sangalli L., Dell’Orto V. (2003). Rumen-protected choline administration to transition cows: Effects on milk production and vitamin E status. J. Vet. Med..

[15] Humer E., Bruggeman G., Zebeli Q. (2019). A meta-analysis on the impact of the supplementation of rumen-protected choline on the metabolic health and performance of dairy cattle. Animals.

[16] Drouillard J.S., Flake A.S., Kuhl G.L. (1998). Effects of added fat, degradable intake protein, and ruminally protected choline in diets of finishing steers. Kans. Agric. Exp. Sta. Res. Rep..

[17] Bryant T.C., Rivera J.D., Galyean M.L., Duff G.C., Hallford D.M., Montgomery T.H. (1999). Effects of dietary level of ruminally protected choline on performance and carcass characteristics of finishing beef steers and on growth and serum metabolites in lambs. J. Anim. Sci..

[18] Bindel D.J., Drouillard J.S., Titgemeyer E.C., Wessels R.H., Loest C.A. (2000). Effects of ruminally protected choline and dietary fat on performance and blood metabolites of finishing heifers. J. Anim. Sci..

[19] Fernández C., Gallego L., Lopez-Bote C. (1998). Effect of betaine on fat content in growing lambs. Anim. Feed Sci. Technol..

[20] Li H., Wang H., Yu L., Wang M., Liu S., Sun L., Chen Q. (2015). Effects of supplementation of rumen-protected choline on growth performance, meat quality and gene expression in longissimus dorsi muscle of lambs. Arch. Anim. Nutr..

[21] Lobley G.E., Connell A., Revell D. (1996). The importance of transmethylation reactions to methionine metabolism in sheep: Effects of supplementation with creatine and choline. Br. J. Nutr..

[22] Dodson M.V., Hausman G.J., Guan L., Du M., Rasmussen T., Poulos S.P., Mir P., Bergen W.G., Fernyhough M.E., McFarland D.C. (2010). Lipid metabolism, adipocyte depot physiology and utilization of meat animals as experimental models for metabolic research. Int. J. Biol. Sci..

[23] White H.M., Richert B.T., Latour M.A., Valenzuela Baez R. (2013). Impacts of nutrition and environmental stressors on lipid metabolism. Lipid Metabolism.

[24] Fouad A.M., El-Senousey H.K. (2014). Nutritional factors affecting abdominal fat deposition in poultry: A review. Asian-Australas. J. Anim. Sci..

[25] Dawson R.M.C., Grime D.W., Lindsay D.B. (1981). On the insensitivity of sheep to the almost complete microbial destruction of dietary choline before alimentary-tract absorption. Biochem. J..

[26] Robinson B.S., Snoswell A.M., Runciman W.B., Upton R.N. (1984). Uptake and output of various forms of choline by organs of the conscious chronically catheterized sheep. Biochem. J..

[27] Robinson B.S., Snoswell A.M., Setchell B.P. (1987). The enterohepatic recycling of bile choline in sheep. Comp. Biochem. Physiol..

[28] Francisco A.E., Santos-Silva J.M., Portugal A.P.V., Alves S.P., Bessa R.J.B. (2019). Relationship between rumen ciliate protozoa and biohydrogenation fatty acid profile in rumen and meat of lambs. PLoS ONE.

[29] Wildeus S., Turner K.E., Collins J.R. (2005). Growth performance of Barbados Blackbelly, Katahdin and St. Croix hair sheep lambs fed pasture- or hay-based diets. Sheep Goat J..

[30] Latimer J.G., Association of Official Analytical Chemists (2019). Official Methods of Analysis of AOAC International.

[31] American Meat Science Association, National Cattlemen’s Beef Association (U.S.), National Pork Producers Council (U.S.) (2001). United States Standards for Grades of Beef, Veal, Pork and lamb Carcasses. Meat Evaluation Handbook.

[32] Li F., Hitch T.C.A., Chen Y., Creevey C.J., Guan L.L. (2019). Comparative metagenomic and metatranscriptomic analyses reveal the breed effect on the rumen microbiome and its associations with feed efficiency in beef cattle. Microbiome.

[33] Dong L., Jin Y., Cui H., Yu L., Luo Y., Wang S., Wang H. (2020). Effects of diet supplementation with rumen-protected betaine on carcass characteristics and fat deposition in growing lambs. Meat Sci..

[34] Wang P., Drackley J.K., Stamey-Lanier J.A., Keisler D., Loor J.J. (2014). Effects of level of nutrient intake and age on mammalian target of rapamycin, insulin, and insulin-like growth factor-1 gene network expression in skeletal muscle of young Holstein calves. J. Dairy Sci..

[35] Banskalieva V., Puchala R., Goetsch A.L., Sahlu T. (2005). Effects of ruminally protected betaine and choline on net flux of nutrients across the portal-drained viscera and liver of meat goat wethers consuming diets differing in protein concentration. Small Rumin. Res..

[36] Cavender C.P., Turley S.D., Dietschy J.M. (1995). Sterol metabolism in fetal, newborn, and suckled lambs and their response to cholesterol after weaning. Endocrinol. Metab..

[37] Turley S.D., Burns D.K., Dietschy J.M. (1998). Preferential utilization of newly synthesized cholesterol for brain growth in neonatal lambs. Endocrinol. Metab..

[38] Bunch T.D., Evans R.C., Wang S., Brennand C.P., Whittier D.R., Taylor B.J. (2004). Feed efficiency, growth rates, carcass evaluation, cholesterol levels and sensory evaluation of lambs of various hair and wool sheep and their crosses. Small Rumin. Res..

[39] Ayala-Monter M.A., Hernández-Sánchez D., González-Muñoz S., Pinto-Ruiz R., Martínez-Aispuro J.A., Torres-Salado N., Herrera-Pérez J., Gloria-Trujillo A. (2019). Growth performance and health of nursing lambs supplemented with inulin and *Lactobacillus casei*. Asian-Australas. J. Anim. Sci..

[40] De Veth M.J., Artegoitia V.M., Campagna S.R., Lapierre H., Harte F., Girard C.L. (2016). Choline absorption and evaluation of bioavailability markers when supplementing choline to lactating dairy cows. J. Dairy Sci..

